# The Rhodamine Isothiocyanate Analogue as a Quorum Sensing Inhibitor Has the Potential to Control Microbially-Induced Biofouling

**DOI:** 10.3390/md18090484

**Published:** 2020-09-22

**Authors:** Yu Song, Shengjie Zhang, Yanhua Zeng, Jianming Zhu, Xiaopeng Du, Zhonghua Cai, Jin Zhou

**Affiliations:** 1 Shenzhen Public Platform for Screening & Application of Marine Microbial Resources, Shenzhen International Graduate School, Tsinghua University, Shenzhen 518055, China; songy17@tsinghua.org.cn (Y.S.); zhang-sj18@mails.tsinghua.edu.cn (S.Z.); zeng.yanhua@sz.tsinghua.edu.cn (Y.Z.); 19b929041@stu.hit.edu.cn (J.Z.); du.xiaopeng@sz.tsinghua.edu.cn (X.D.); caizh@sz.tsinghua.edu.cn (Z.C.); 2 The Department of Earth System Science, Tsinghua University, Beijing 100084, China; 3 Institute for Ocean Engineering, Tsinghua University, Beijing 100084, China

**Keywords:** biofouling, anti-biofilm, quorum sensing inhibitor, multiple-species, rhodamine isothiocyanate analogue

## Abstract

Quorum sensing inhibitors (QSIs) have been proven to be an innovative approach to interfering with biofilm formation, since this process is regulated by QS signals. However, most studies have focused on single-species biofilm formation, whereas studies of the effects of signal interference on the development of multispecies biofilm, especially in the natural environment, are still lacking. Here we develop and evaluate the anti-biofilm capability of a new QSI (rhodamine isothiocyanate analogue, RIA) in natural seawater. During the experiment, biofilm characteristics, microbial communities/functions and network interactions were monitored at 36, 80, and 180 h, respectively. The results showed that the biomass and 3D structure of the biofilm were significantly different in the presence of the QSI. The expression of genes involved in extracellular polysaccharide synthesis was also downregulated in the QSI-treated group. Dramatic differences in microbial composition, β-diversity and functions between the RIA-treated group and the control group were also observed, especially in the early stage of biofilm development. Furthermore, co-occurrence model analysis showed that RIA reduced the complexity of the microbial network. This study demonstrates that rhodamine isothiocyanate analogue is an efficient QS inhibitor and has potential applications in controlling biofouling caused by multispecies biofilm, especially in the early stage of biofouling formation.

## 1. Introduction

Biofilms are crucial in a wide range of marine events and could lead to microbial-induced biofouling [1]. The biofouling process is closely related to microorganisms and their metabolic products, including organic and inorganic acids, extracellular polymeric substances (EPS), proteins, hydrogen sulfide and ammonia [2]. The biofouling phenomenon leads to huge economic losses and numerous ecological issues worldwide [3]. In the shipping industry, biofouling induces yard platform damage, metal corrosion and physical damage of ship hulks, increases energy consumption and drag force, reduces heat transfer efficiency, and also introduces alien species from prior destinations [4]. Therefore, biofouling has drawn more and more interest and become a focal point of novel and multidisciplinary research.

Early antifouling strategies included physical cleaning (this strategy involves extra maintenance costs and requires extra labor) and the use of oxidizing agents such as ozone, hydrogen peroxide, chlorinated compounds (i.e., chlorine or bleach), detergents (e.g., sodium dodecylsulfate and benzalkonium chloride), antibacterial compounds (e.g., metal and cooper salts), diverse biocides such as tributyltin, and aldehydes (e.g., gluteraldehyde and formaldehyde) [5,6]. All these compounds have toxicity, which could destroy the ecological balance and therefore might cause environmental and health problems. Fortunately, with the development of modern biological technology, some bio-remedies have attracted more and more attention and are widely used for long-term biofouling treatment based on their unique advantages, including safety, environmental friendliness, substitutability, and sustainability [7,8].

Recent studies have elucidated the involvement of quorum sensing (QS, bacterial cell-to-cell communication) in biofilm formation, reporting that bacteria can communicate with each other via small signal molecules called autoinducers [6]. These signal molecules can bind to cognate receptors to form a complex, which binds to a promoter to trigger the initiation of biofilm formation [9]. Based on this, efficient anti-fouling strategies need to consider the formation and characteristics of biofilms. It is well known that biofilm requires intact cell–cell communication (such as the QS mechanism) for its initiation and maturation [6,9]. Therefore, abolishing the QS mechanism using quorum sensing inhibitors (QSIs) can efficiently prevent the development of bacterial biofilms as well as the subsequent biofouling [10].

Various types of QSIs have been isolated from natural resources [11], including algae, plants, bacteria, and fungi. These QSIs can hinder biofilm formation and the subsequent development of biofouling [12,13,14,15]. This strategy has been acknowledged to be a sustainable antifouling method and has been investigated widely using model bacterial systems. Yeon et al. (2009) showed that the QSI of porcine kidney acylase I at 10 mg/L can significantly decrease the concentrations of signal molecules in the biofilm and effectively suppress biofouling [16]. In addition, another QSI, halogenated furanone, isolated from red marine algae, has been identified as a potent antagonist of Gram-negative bacteria [17]. A long-chain fatty aldehyde, pentadecanal, was observed to act against *Staphylococcus epidermidis* biofilm formation [18]. A 2-sufonylpyrimidine was demonstrated to effectively inhibit *Pseudomonas aeruginosa* biofilm formation [19]. We have also developed a new QSI (rhodamine isothiocyanate analogue), which demonstrates significant anti-biofilm ability on *P. aeruginosa* strain PAO1 under laboratory conditions [20]. However, most studies exploring the role of QSI have focused on the formation of single-species biofilm using a model bacterium (e.g., *Staphylococcus aureus* or *Pseudomonas aeruginosa*) [16,21], and thus far there has been no study on natural biofilm formation involving complex communities. Furthermore, the complex natural environment cannot be exactly imitated by the laboratory-scale systems. Hence, it is essential to address the environmental perspectives in order to better evaluate the anti-biofouling efficiencies of biological methods.

In this study, following our previous work [20], we further investigated the anti-biofouling potential of rhodamine isothiocyanate analogue in the natural marine environment. We designed a series of semipermeable dialysis bags and hung them under the surface-water. The biofilm profiles, EPS functional genes, and the development of the biofilm community structure were assessed at three time-points (36 h, 80 h and 180 h). The aim of the current study was to compare the structure and composition of the biofouling microbiome under QSI interference using fluorescence microscopy and microbial 16S rRNA sequencing analysis. We aimed to supply natural-scale evidence about the anti-biofilm efficiency of rhodamine isothiocyanate analogue, as well as supplying possible natural resources for developing anti-biofouling agents.

## 2. Results

### 2.1. Effect of QSI on Biofilm Formation

**Figure 1:**
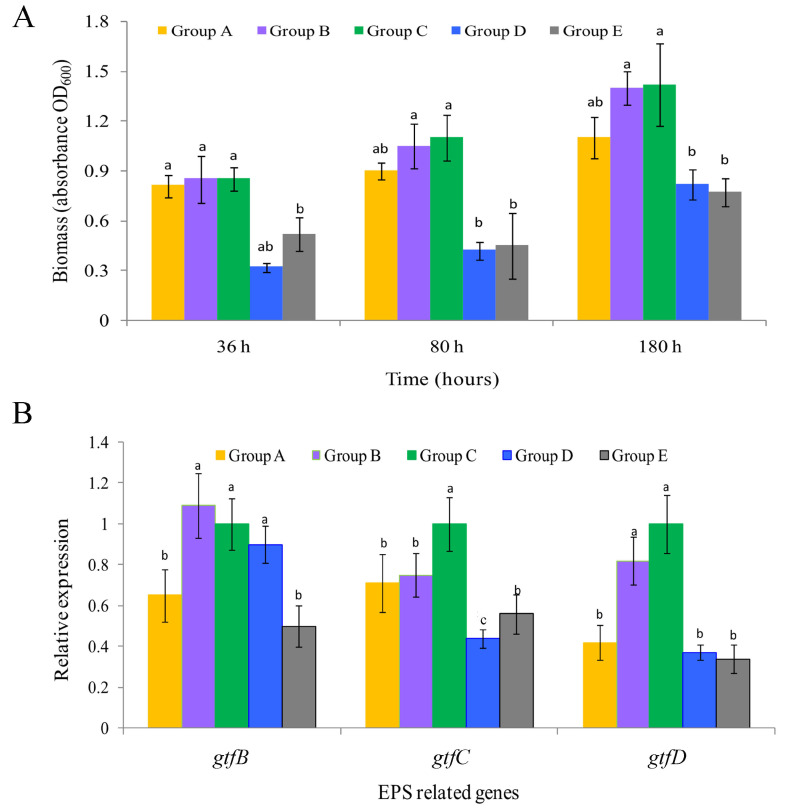
(**A**) The biomass of biofilm under different treatments across three different time-points. The data are shown as the mean ± standard error of three independent experiments performed in duplicate. (**B**) Changes in glycosyltransferase genes (*gtfB*, *gtfC* and *gtfD*) expression related to extracellular polymeric substance (EPS) formation of the 36-h old biofilms. Values are shown as the mean ± standard error. The different letters (a, b and c) on the column indicate statistically significant differences among the different treatment groups at the *p* < 0.05 level.

In the control group (group C), a clear surface biofilm was formed from 36 h, and the biomass continued to increase until 180 h (Figure 1A). When the membrane was exposed to the QSI, both the positive control group (furanone, group E) and the experimental groups treated with QSI active extracts (groups A and D) showed a significant inhibitory effect on biofilm development (*p* < 0.05). The most significant inhibition was observed in group D, in which the QSI caused the biomass to decrease by 62.3% compared to the control group (36 h) (*p* < 0.05). In the later stage of biofilm formation (80 and 180 h), the biomass of the experimental groups was also significantly lower than that of the control group (*p* < 0.05). The apparent results of biofilm morphology are shown in Figure S1. A qualitative description method was used to recode the changes of biomass signs in each group, i.e., strong (+++), medium (++), and weak (+), and the results revealed a considerable reduction in biomass in the QSI-treated groups.

**Figure 2:**
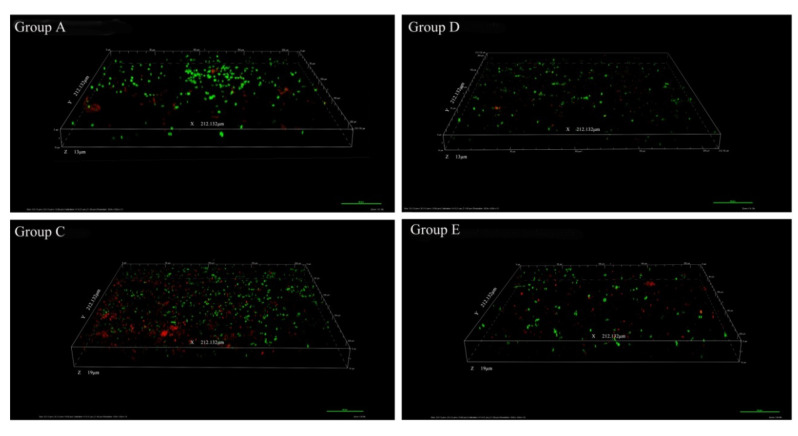
Confocal laser scanning microscopy (CLSM) photographs of the natural biofilm architecture, in the presence or absence of the quorum sensing inhibitor (QSI) extracts, taking 36 h as an example (200×). The images shown represent the CLSM z-stack 3D images of biofilms treated with extracts from *Vibrioalginolyticus* H12 (group A), blank control seawater only (group C), rhodamine isothiocyanate analogue (group D), and the positive control furanone (group E). It should be noted that the results of group B and control group C were similar, so we ignored the image of group B.

CLSM(confocal laser scanning microscope) z-stack 3D images precisely evaluated the structures of biofilms at different time-points. Taking 36 h treatment as an example, relatively thicker and denser biofilms were formed in the control group (C) with more cells compared to those in the experimental groups (Figure 2). In the control group (C), some attached bacteria had connected into flakes with a tendency to form biological film. In contrast, the biofilms treated with inhibitors (such as extracts of *Vibrioalginolyticus* H12 (group A) or rhodamine isothiocyanate analogue (group D)) were mostly scattered and sporadically distributed, showing a disrupted surface topology profile of the biofilm under QSI treatment. In addition, after treatment of biofilms with QSI for 36 h, the expression of genes related to EPS synthesis (*gtfB*, *gtfC* and *gtfD*) were significantly downregulated by 25.1–66.2% in QSI-treated groups compared with the control group (*p* < 0.05) (Figure 1B).

### 2.2. Effect of QSI on the Biodiversity of Natural Biofilm

**Figure 3:**
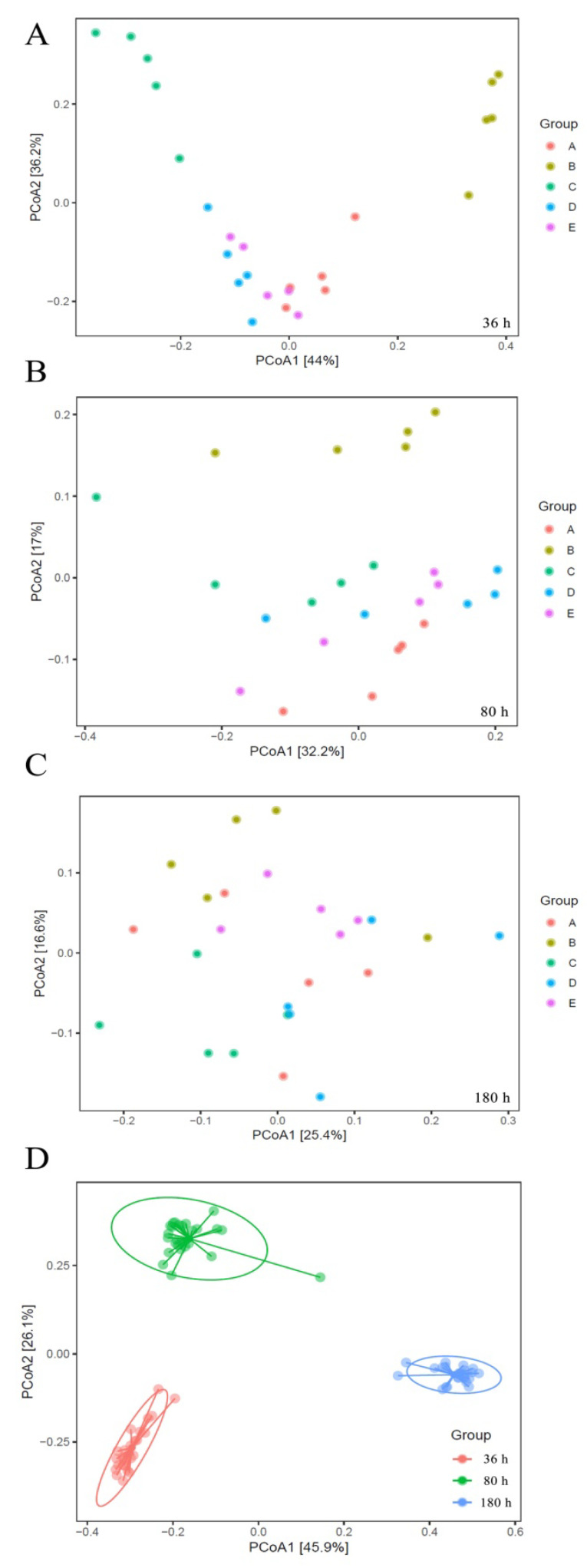
Principal coordinate analysis (PCoA) plots of dissimilarities among biofilm-forming bacterial communities from three different time-points (**A**–**C**). Picture (**D**) is the cluster of various groups at different time-points.

Considering α-diversity, both the QSI-producer and QSI substance (groups A, B, D and E) did not cause significant changes (*p* > 0.05) in the Chao 1, Shannon, or Simpson indices (Table S1). The α-diversity was similar with that of the blank control group C. Considering β-diversity, principal coordinate analysis (PCoA) can be used to investigate the characteristics of the community changes. The presence of QSI (groups A and D) dramatically affected the biodiversity (*p* < 0.05), and there were significant differences between QSI-treated samples and control samples in the early stage of biofilm development. The bacterial communities of the biofilm in group B (QSI-producer treatment) and groups A and D (QSI-substance treatment) clustered away from group C (blank control) (Figure 3A). However, the separation of biofilm communities did not vary significantly between all the groups in the later stage of biofilm development (80 and 180 h) (Figure 3B,C), suggesting that the influence of QSI on β-diversity is no longer evident in the maturity stage of biofilm formation. In addition, compared with the inter-group data, a clearer cluster was observed in the PCoA plot along the timescale (Figure 3D), implying that the time factor has a larger influence on the β-diversity of biofilm.

### 2.3. Effect of QSI on the Composition of Natural Biofilm

The statistical analysis showed a significant difference in biofilm-associated bacterial communities among QSI treated groups (groups A, B, D, and E) and the control group (*p* = 0.008 − 0.031) at the early biofilm stage, especially at 36 h (Table 1). Entering the post-biofilm stage (180 h), the statistical difference between the QSI-added groups (D and E) and the control group was still remarkable, with *p* values of 0.013 and 0.008, respectively.

**Figure 4:**
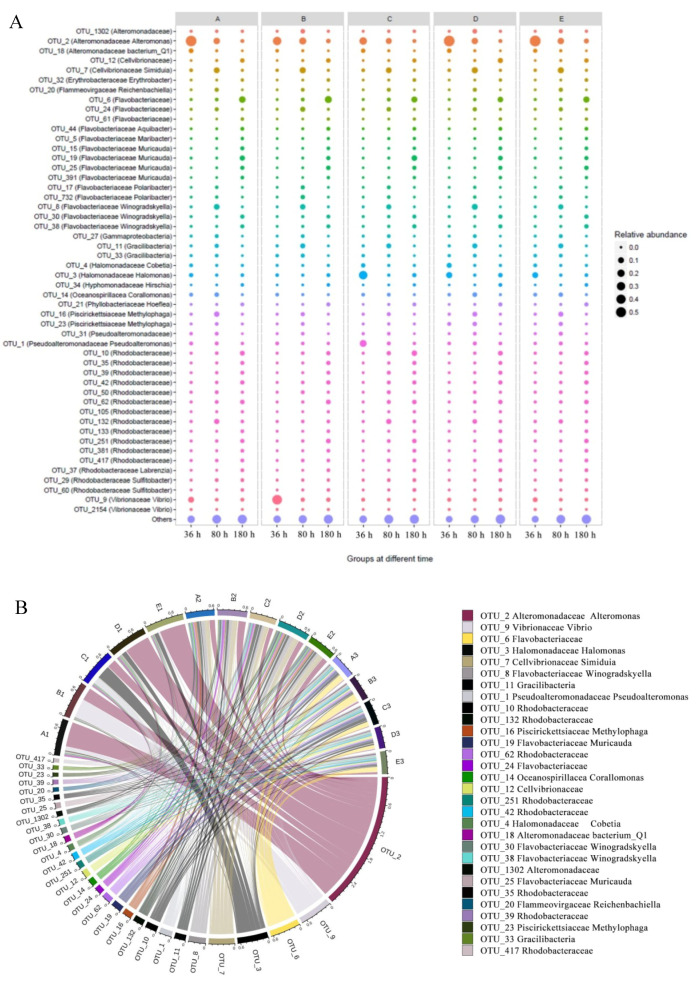
(**A**) Circos diagrams illustrating microbial composition across 5 groups treated across 3 time-points at the genus level. Left and right semicircles represent different genus and various samples, respectively. The main taxa operational taxonomic units (OTUs) are across the five groups across the three time-points. The thickness of the line represents the relative abundance of bacteria. (**B**) Relative abundance of biofilm-associated bacterial communities sorted taxonomically at the genus level for the five groups at different time-points. The sizes of the circles represent the relative abundance of bacteria.

The detailed changes in community compositions were also analyzed. Bacterial communities in biofilm were dominated by Proteobacteria, Bacteroidetes, Gracilibacteria, Planctomycetes, and Cyanobacteria at the phylum level (Figure S2A). At the family level, the addition of QSI led to significant changes in the community composition during the experiment, especially in the first 36 h of biofilm formation (Figure S2B). Specifically, the abundance of Halomonadaceae was found to be high (up to 15%) in the control group, where as it was significantly inhibited to less than 2% in the QSI-treated groups (such as A, D, and E) (Figure 4A). In addition, the relative abundance of Alteromonadaceae and Flavobacteriaceae significantly increased by more than 2.5 times after treatment with QSI. The changes of community in group D was much more significant than those in group E (positive control).

Zooming in at the genus level, univariate analysis was performed on some representative taxa to show clearer changes. As shown in Figure 4B, *Pseudoalteromonas* and *Halomonas* were significantly less abundant in the QSI-treated biofilm microbiomes (*p* < 0.05). Specifically, after QSI treatment, *Pseudoalteromonas* decreased more than five-fold, with the relative abundance decreasing from 15.3% to 2.9%. In contrast, after QSI treatment (such as in groups D and E), the relative abundance of *Alteromonas* increased significantly (more than three-fold) over the course of the experiment. It is speculated that QSI could inhibit the colonization of QS strains, and thereby inhibit the development of biofilm. Other alterations were also observed among the QSI treated groups and control, such as *Rhodobacteraceae* and *Flacobacteriaceae*.

To elucidate whether the biofilm inhibition is related to environmental parameters, the temperature, salinity, pH, and nutrients (NO_3_^−^, PO_4_^3−^, NH_4_^+^, and TOC (total organic carbon)) were examined. Results showed that the temperature, salinity and pH ranges were 24.35–26.59 °C, 32.33–32.98‰, and 7.99–8.01, respectively (Table 2). The ranges of PO_4_^3−^, NO_3_^−^, and NH_4_^+^ were 1.28–1.72 μmol/L, 13.27–18.63 μmol/L, and 22.19–31.43 μmol/L, respectively. The concentration of TOC ranged from 2.86 to 4.97 mg/dm^3^. However, for all groups, no significant changes were observed for all these factors during the whole experimental period. The main influencing factors on biofilm microorganisms were temperature, TOC, PO_4_^3−^, and NH_4_^+^ (Figure S3).

### 2.4. Network Analysis of Biofilm Microorganisms under QSI Treatment

**Figure 5:**
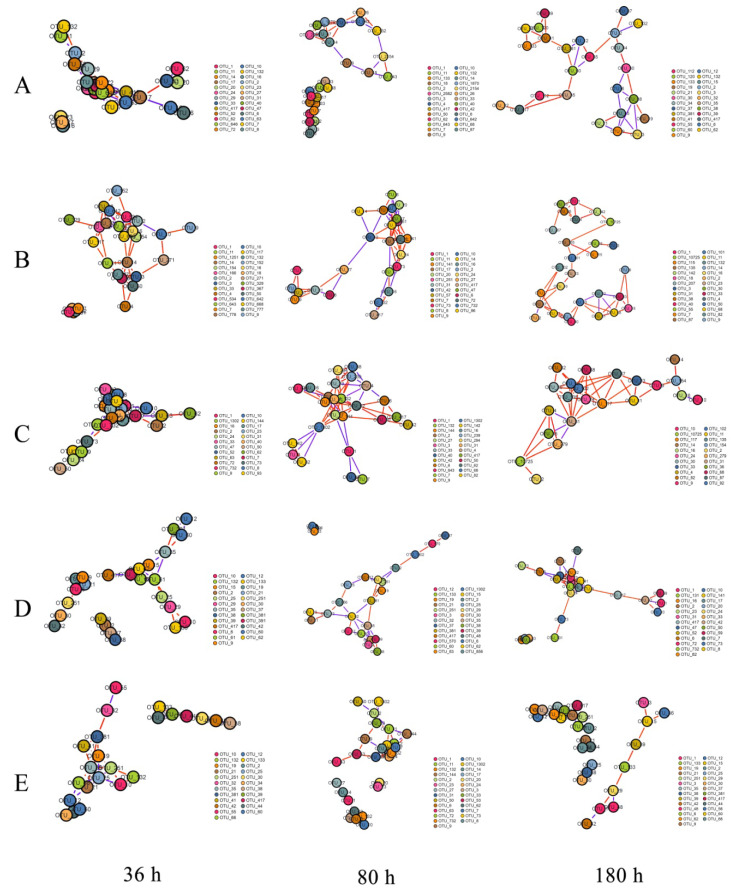
Species correlation network based on the relative abundance of species in the samples in the OTU table. The red and blue edges represent the positive correlation (PC) and negative correlation (NC) between pairwise OTUs, respectively. The size of nodes is proportional to the number of connections, and different colors of nodes represent different modules. (**A**) extracts from *V. alginolyticus* H12, (**B**) bacterial cultures of *V. alginolyticus* H12, (**C**) blank control, (**D**) QSI compound rhodamine isothiocyanate analogue, and (**E**) positive control furanone, across three time-points (36, 80, and 180 h).

The community networks were constructed and the relative abundance of bacterial operational taxonomic units (OTUs) (the main OTUs belonging to Alphaproteobacteria and Gammaproteobacteria) at the family level formed the nodes; the links were mainly represented by Alteromonadaceae, Rhodobacteraceae, Vibrionaceae, and Halomonadaceaein the presence of QSI in groups A, B, and D, whereas the nodes of group E were represented by Flavobacteriaceae, Rhodobacteraceae and Gracilibacteria. These species mainly came from 11 OTUs, including OTU 1, 2, 4, 9, 11, 14, 19, 30, 38, and 39. In the network (Figure 5), nodes represent bacterial OTUs and edges represent correlations between pairwise OTUs. The major topological properties (the average connectivity, clustering coefficients, and modularity) are shown in Table 3. Compared with the control group C, the clustering coefficient and network connectivity were significantly decreased 10.95–45.33% in QSI-treated groups A, B, and D. In addition, there was a relatively larger proportion of negative correlations and looser module structures (modularity) in QSI-treated samples compared with the control. These results showed that QSI, especially rhodamine isothiocyanate analogue, reduced network complexity and co-occurrence patterns, which further affected the stability and environmental buffering capacity of the biofilm microbial community.

### 2.5. Functional Profiles of Biofilm Microorganisms under QSI Treatment

**Figure 6:**
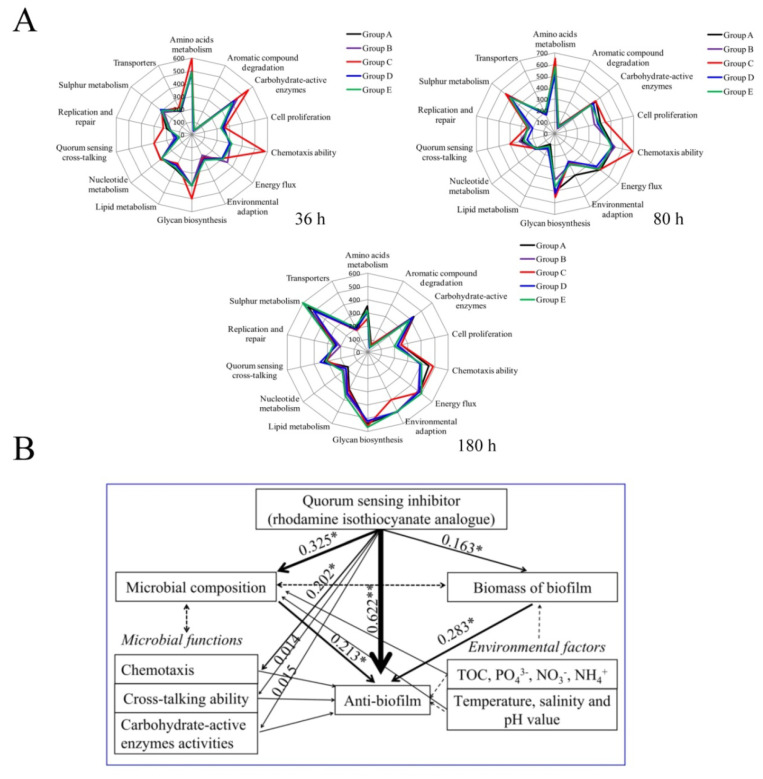
(**A**) the predicted functions of biofilm-associated microorganisms in different groups at various time-points. (**B**) structural equation modeling showing the effects of QSI and environmental properties on metabolic activities, bacterial community structure, and anti-biofilm potential in natural conditions. The width of arrows indicates the strength of significant standardized path coefficients, black solid lines indicate positive effects, and paths with uncertain coefficients are presented as dotted lines. The single asterisk (*) and double asterisk (**) indicates a significant difference on each other at *p* < 0.05 and *p* < 0.01 level, respectively.

We evaluated the metabolic potential if QSI-induced changes in community structure and alteration in community functioning co-occurred. Based on the functional genes detected, the metabolic potential of bacterial communities differed in various groups. Carbohydrate, amino acid, and glycolipid metabolisms were the main pathways in all groups, whereas in QSI-treated groups, some metabolic pathways were enhanced, such as environmental adaptation ability (Figure 6A). Unlike the environmental adaptation genes, QS functional genes were significantly decreased in QSI-treated groups, and this inhibition was particularly pronounced at the beginning of biofilm formation (36 h). The proportion of QS functional genes in the control group was 5.7%, whereas the proportion was 3.2%, 2.7%, and 2.3% in group A (QSI active extracts treated), group B (QSI-positive strain treated), and group D (rhodamine isothiocyanate analogue treated), respectively. Like the cross-talking genes, QSI remarkably reduced the chemotaxis of bacteria. For example, the abundance of genes involved in chemotaxis in group D decreased 2.04-fold compared with the control group (at 36 h, *p* < 0.05).

### 2.6. Linkage of QSI to Biofilm Inhibition

The effects of QSI and physicochemical parameters on microbial community structure, metabolic functions, and biofilm inhibition were evaluated using structural equation modeling (SEM) (Figure 6B). Results showed that environmental factors (such as TOC (total organic carbon), NH_4_^+^, and PO_4_^3−^) had positive effects on biofilm-associated microbial composition. In addition, the SEM analysis revealed that QSI significantly affected microbial community structure and metabolism potential, which directly or indirectly influence anti-biofilm ability (R^2^ = 0.622 and 0.325, respectively). These results suggest that QSI is a main contributor to the anti-biofilm phenotype.

## 3. Discussion

In this study, we designed a dumpling-shaped semipermeable membrane bag, facilitating prolonged survival and active life of the QSI bacterium. Using this bag, the anti-biofouling potential of QSI-like compound (groups A and D) was evaluated. As shown in Figure 1A, we observed a significant decrease in the biomass of biofilm, indicating that the QSI compounds can inhibit the development of biofilm. The confocal microscopy results showed that the 3D structures of groups A, D, and E were looser than those of the control (group C) (Figure 2), which provides evidence that the rhodamine isothiocyanate analogue has the potential to interfere with biofouling. The underlying mechanism may be related to EPS, a key component associated with biofilm formation [22]. Indeed, significantly lower expression levels of EPS related genes (*gtfB*, *gtfC*, and *gtfD*, which catalyze glycosidic bond formation and participate in EPS biosynthesis) were observed in groups A and D compared to those in control group (C) (*p* < 0.05) (Figure 1B), which may explain the biofilm blocking effect of QSI [16,23]. In addition, as shown in Figure S1, the thickness and roughness of the biofilm were significantly decreased in the QSI-treated group (D) compared with the control group (C), indicating that QSI can affect the arrangement of cells in the biofilm and eventually slow down the biofilm’s formation [24].

The microbial diversity and composition were analyzed to elucidate the effects of QSI on the community structure and dynamic process of the biofilm. Although there was no statistically significant difference, we observed a relatively higher Chao 1 index in QSI treated groups (B and D) compared with the control group (C) (Table S1). A possible explanation for this is the “intermediate disturbance hypothesis”, i.e., with the disturbance of an intermediate ecology, the local species diversity is maximized [25]. Our results matched the “intermediate disturbance hypothesis”, and the relative higher α-diversity resulting from QSI treatments may support this conjecture. As for the microbial composition of biofilm, as shown in Table 1, during the whole experimental period, there were significant differences between QSI-treated groups (A, D, and E) and the control group (C) (*p* < 0.05 or 0.01). These results suggest that QSI has the capability to modulate the proportions of certain microbial groups in the natural environment. Additional evidence can be found from the PCoA results. Figure 3A shows the potential separation between the treatment groups (A, D, and E) and the control group (C) as early as 36 h post-treatment. Moreover, the relative percentages of *Pseudoalteromonas* and *Halomonas* were significantly decreased in two QSI-treated groups (A and D) (*p* < 0.05) (Figure 4A). These patterns indicate that QSI can influence the biofilm-associated microbial community structure and change the species populations. Similar to our results, a previous study showed that after several days of QSI treatment, a shift of community-dominating species from *Oceanospirillales* to *Rhodobacterales* was observed [26]. These results showed that QSI can regulate the relative abundances of bacteria and alter the biodiversity of a symbiotic community, thereby disturbing the “normal flora”, and eventually leading to biofilm inhibition [27]. However, there was no significant difference between the experimental groups (A, D, and E) and the control group (C) at 180 h, suggesting that QSI exhibits biological activity during the early stage of biofilm formation, while only having limited ability at the later stage when the biofilm is reaching the maturity stage.

The pioneer primary colonizers play important roles at the initial stage of biofouling, because they supply nutrients or related niches for secondary colonizers to thrive [28,29]. In this study, we observed that the abundance of some pioneer bacteria like *Pseudoalteromonas* and *Halomonas* were relatively lower in the QSI groups (A and D), but higher in the control group (C) (Figure 4B). Specifically, the relative abundance of *Pseudoalteromonas* in groups A and D decreased more than five-fold after QSI-treatment, compared with that of the control group (group C). Numerous reports have shown that biofilm formation is closely associated with *Pseudoalteromonas* (a typical bacterium that relies heavily on QS), which could regulate microbial population and help other bacteria colonize [30,31,32]. Like *Pseudoalteromonas*, *Halomonas* is also a QS producer that is sensitive to QSI. Previous reports have shown that *Halomonas* displays swarming and twitching motility and can produce polysaccharides, and therefore they are able to flourish during the late phase of biofilm formation following the initiation of biofilm formation as pioneer bacteria [30,33]. The decrease in *Halomonas* further confirmed that rhodamine isothiocyanate analogue has the potential to reduce the relative abundance of QS-related microorganisms and subsequently to affect the biofilm development pattern.In order to investigate the influence of QSI on ecological interactions among the biofilm-associated multispecies communities [34], network analysis was carried out. The control group (C) had relatively complex interactions, whereas the QSI-treated group (D) had the loosest link (Figure 5), indicating that QSI decreased the network complexity of biofilm microorganisms. The network complexity is critical for microbial homeostasis and ecological buffer capability [35,36,37,38]. During the pre-stage of biofilm formation, the adhesion behaviors of individual bacteria are dynamic. Under these circumstances, bacterial community structures are highly unstable [39]. Therefore, the disruption of microbial network complexity by QSI can alter the biofilm’s microbial interactions and subsequently affect the initiation of biofilm formation [35,37]. In addition, the percentage of positive relationships in the network was also affected by QSI. Positive associations indicate the occurrence of prevalent mutualism or commensalism [40]. In this study, relatively lower positive interactions in the QSI groups imply disruption of cooperative behaviors, such as bacterial co-colonization and co-aggregation within biofilm communities during the initial adhesion process [39], which could be conducive to the formation of biofilms. Taking together, the network results suggest that QSI can alter the network topological profile in biofilm-associated species and therefore hinder the biofilm formation (Figure 5, Table 3), which provides knowledge bridging the gap between the alteration of bacterial interactions and biofilm formation in the presence of QSIs [36,38].

Considering that the microbiome structure is closely related to their functions, we used PICRUSt software to predict the functional changes under QSI treatment. Results showed that the main biological functions associated with biofilm formation included carbohydrate and protein metabolism, as well as polysaccharide biosynthesis (Figure 6A), which is consist with the results of Douterelo [41]. Among the functional groups detected, the most significant one is chemotaxis. The QSI-treated groups A, B, and D showed significantly lower relative abundance related to chemotaxis than the control group, which was only half of that in the control (*p* < 0.05) (Figure 6A). Chemotaxis action participates in the establishment of symbiotic relationships and plays a crucial role in shaping biofilm microbial composition [42]. Based on the fact that the biofilm surface environment is characterized by strong gradients of chemical cues and organic molecules [43], chemotaxis and motility are important behaviors favoring colonization of bacteria. In addition, we found that the carbohydrate-active enzymes (CAZy) in QSI groups (A and D) were significantly decreased compared with the control group (C) at 36 h, suggesting that CAZy is an effector in biofilm formation. Previous reports showed that CAZy deficiency can inhibit biofilm formation [44]. In this study, QSI downregulated CAZy-related genes. Under these conditions, the microbial surface energy was decreased, which led to the limitation of biofilm formation [45]. Additionally, it is worth mentioning that in QSI-exposed groups, genes related to cross-talking (such as quorum sensing) were remarkably declined. The QS signal factors are able to regulate microbial concentration and provide fitness advantages to microorganisms, and this can be a strategy to survive in various environments [6]. Furthermore, the roles of QS in multi-species biofilm communities are the same, in spite of the complexities of these microbiomes [46]. These results suggest that QSI can affect microbial metabolic activity and the development pattern of biofilm.

## 4. Materials and Methods

### 4.1. Experimental Design

**Figure 7:**
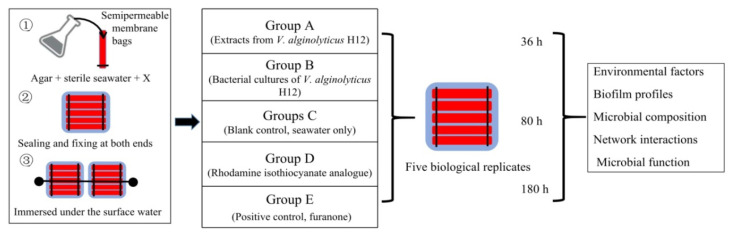
Sketch of the experimental design used in this work. There were three test groups—group A, extracts from *V. alginolyticus* H12; group B, bacterial cultures of *V. alginolyticus* H12; and group D, rhodamine isothiocyanate analogue. The positive control was group E (furanone). Additionally, the blank control group (group C) was only exposed to the seawater, without adding any substances. Agar was mixed with sterile seawater, and the compounds were added into the dialysis bag according to the experimental setup. The ends of the bags were closed using clips. The bags were immersed in seawater to a depth of approximately 50–100 cm. Each group had five parallel samples, and 3 time-points were set: 36 h, 80 h, and 180 h.

The crude extracts and purified active compounds (rhodamine isothiocyanate analogue) from QSI strain *Vibrio alginolyticus* H12 were prepared as previously described [20]. To evaluate the anti-biofilm and antifouling properties of rhodamine isothiocyanate analogue, the bacterial cultures and purified active extracts (rhodamine isothiocyanate analogue) of strain H12 were mixed with sterilized agar, respectively, and encapsulated in a semipermeable membrane bag. As shown in Figure 7, a total of five groups were included—group A, extracts of *V. alginolyticus* H12; group B, bacterial cultures of *V. alginolyticus* H12; group C, blank control (seawater only); group D, rhodamine isothiocyanate analogue; and group E, positive control (furanone). Furanone is a typical QSI purchased from Sigma-Aldrich (St. Louis, MO, USA), and 1 mM of furanone was used in the experiment because furanone below this concentration is known to be less toxic to cells [47]. For all treatment groups, the final concentrations of QSI substance and QSI-producers (i.e., bacterium) in the bags were 1 mM and 1.0 × 10^5^ CFU/mL (colony forming units per milliliter), respectively. All the semipermeable membrane bags were immersed under the surface of seawater (0.5–1 m). Each treatment had five biological replicates.

### 4.2. Measurement of Environmental Parameters

The samples were harvested at three time-points (36 h, 80 h, and 180 h) of the experimental cycle. The surface of semipermeable membrane bags was monitored for any signs of biofilm formation, and photographs of them were taken. Temperature, salinity, and pH values were measured using a conductivity-temperature-depth (CTD) profiler (SBE19, Sea-Bird Scientific, Bellevue, WA, USA). The nutrient factors—ammonium nitrogen (NH_4_^+^), nitrate nitrogen (NO_3_^−^), and dissolved inorganic phosphorus (PO_4_^3−^)—were measured using a Discrete Chemistry Analyzer (CleverChem Anna, Germany). The total organic carbon (TOC) was measured as previously described [48], using an Apollo 9000 Total Organic Carbon Analyzer (Teledyne Instruments Tekmar, Mason, OH, USA).

### 4.3. Detection of Biofilm Profile

The biomass of the biofilm was quantified using crystal violet staining at each sampling time-point, as previously described [20]. Briefly, the membrane was incubated in 0.1% crystal violet (CV) solution for 30 min, washed, and then the CV was solubilized from the stained cells with 95% ethanol. The absorbance of CV was measured at 580 nm using a UV-Vis Spectrophotometer (Tecan, Wien, Austria) and the biomass of biofilm was quantified.

To explore the structure of biofilms, cells were grown in natural seawater for 36, 80, and 180 h, washed, and then stained with 5 μM PI and 5 μM SYTO9 for 15 min in the dark. The cells on one side were wiped off. The slides were then washed, and the biofilms were observed under a confocal microscope (LSM 710 Zeiss, Weimer, Germany) and quantified using COMSAT software [49,50]. 3D photos of biofilms were generated using a FV10-ASW2.0 Viewer (Olympus, Tokyo, Japan), as described previously [51], and digital images were analyzed using Leica Confocal Software Lite (Leica Microsystems, Wetzlar, Germany).

To detect the related functional genes involved in EPS synthesis, the expression levels of three representative glucosyltransferase genes (*gtfB*, *gtfC*, and *gtfD*) were evaluated. Briefly, the biofilms were homogenized by sonication and the total RNA was extracted using a Trizol RNA isolation kit (Life Technologies, Carlsbad, CA, USA). Reverse transcription and real-time PCR (RT-PCR) were conducted by following the manufacturers’ instructions with primers used previously [52]. The relative expression of each gene was quantified by normalizing to the internal reference 16S rRNA gene, and the relative changes in mRNA level were calculated using the 2^−ΔΔCt^ method [53]. Results are expressed as mean ± SD of samples in triplicate.

### 4.4. Determination of Microbial Communities in Biofilm

From each time-point, the microbial samples collected from the biofilm surface were used to extract DNA using a Fast DNA Spin Kit (mBio, Los Angeles, CA, USA). The extracted DNA was quantified and stored at −20 °C for further use.

Prokaryote 16S rRNA genes were amplified using the following primer pairF515 (5′-GTGCCAGCMGCCGCGG-3′) and R907 (5′-CCGTCAATTCMTTTRAGTTT-3′), which can be used for microbial taxonomic classification [54]. The reactions were performed as follows in a 50 μL volume—pre-denaturation at 95 °C for 3 min; 28 cycles of denaturation at 95 °C for 30 s, annealing at 55 °C for 30 s, elongation at 72 °C for 45 s; and a final extension at 72 °C for 10 min. The PCR products were purified using a QIAquick G01 Extraction Kit (Qiagen, Valencia, CA, USA). Equimolar amounts of PCR products were prepared for Miseq. The sequencing was done by Magigene Technology Co., LTD (Guangzhou, China) on a MiSeq platform (Illumina, San Diego, CA, USA) using a 250bp paired-end sequence read run.

### 4.5. Processing Sequencing Data

Raw sequences were first checked using Mothur and QIIME (Quantitative Insights Into Microbial Ecology), and then trimmed and filtered as described previously [55]. Briefly, sequencing primers were removed from the raw sequence reads. If the sequences had one of the following situations, they were removed—if they were less than 200 nt, with a quality score of lower than 25, were identified as chimeric, or had homopolymeric regions greater than 6 nt. After these low-quality reads were removed, the representative sequences were annotated using a basic local alignment search tool (BLAST) by searching against the Silva database and the Ribosomal Database Project. Then the operational taxonomic units (OTUs) were clustered at 97% identity using the UPARSE tool pipeline (v7.1, http://drive5.com/uparse/) [56]. Singletons resulting from putative sequencing errors or PCR amplification artifacts were removed to avoid artificial inflation of microbial diversity [57]. UCLUST (an algorithm for clustering OTUs data) was used for taxonomic assignments with the SILVA 128 reference database. Besides singletons, OTUs associated with mitochondrion, chloroplast, and unclassified and unassigned sequences were also removed from the dataset. The sequence data were deposited in the NCBI GenBank database with an accession number of SRP265754.

### 4.6. Microbial Composition, Co-Occurrence Pattern, and Functional Prediction Analysis

The 16S rRNA gene sequences were analyzed according to the standard Quantitative Insights Into Microbial Ecology (QIIME) procedure [58]. Hierarchical cluster analysis of Bray–Curtis similarity data and one-way analysis of similarity were performed to investigate similarities or significant differences of microbial community composition. Mothur was applied for calculation of α- and β-diversity indices [58]. Welch’s *t* test was used to analyze the statistically significant differences of taxa abundance [59]. 2STAGE analysis was carried out for comparison of bacterial community structures in the PRIMER package (PRIMER v6, PRIMER-E Ltd., Luton, UK) [60]. Principal components analysis (PCA) was performed to display and compare the compositions of microbial communities using Canoco 5 software V5.02 (http://www.canoco5.com).

Interaction network analysis was conducted to explore the co-occurrence patterns among the biofilm microbes using the maximal information coefficient (MIC) scores of the statistics [61]. To simplify the network, only the top OTUs with higher relative abundance in the microbial community were determined. The network analysis was carried out on the OTUs contributing to 90% difference in bacterial communities among treatments, which was identified through SIMPER. Only strong negative or positive relationships were displayed in the network diagrams to strengthen the key interactions. Next, the networks were visualized using Cytoscape v3.4.0 [62], and the complexity of each network, including the average connectivity, clustering coefficients, and node number, was analyzed using the Network-Analyzer tool [63]. The 16S rRNA gene-based microbial compositions were used to predict the functions of the microbial communities in different groups, and the PICRUSt (v1.0.0) protocol was used to draw inferences from the KEGG (Kyoto Encyclopedia of Genes and Genomes) annotated databases [64].

### 4.7. Statistical Analysis

Significance analysis was performed using SPSS (statistical product and service solutions) 11.0 with one-way analysis of variance (ANOVA). A *p* value < 0.05 was considered a significant difference, and *p* < 0.01 was considered a highly significant difference. For 16S diversity analysis, the related methods used are described in the Section 4.5. Structural equation modeling (SEM) analysis was performed using AMOS 21 software, as described previously [49].

## 5. Conclusions

In summary, this work demonstrates that the QSI (rhodamine isothiocyanate analogue) can effectively inhibit biofilm formation and community structures on a field-scale. Its possible mechanism is to decline the initial colonization of pioneer microorganisms, hinder the expression of EPS functional genes, disturb the co-occurrence pattern of biofilm members, and change some microbial functions, such as chemotaxis action, cross-talking capacity, and CAZy enzyme activities. The SEM results (Figure 6B) provide further evidence that biofilm inhibition tended to be controlled by QSI. This is the first investigation of the anti-biofilm potential of rhodamine isothiocyanate analogue in multiple species under real-life natural conditions. The work presented here suggests that QSI has broad application potential in controlling biofouling. In the future, additional studies are required to develop an ideal method of immobilizing QSI substances into a cartridge system for eventual scaling, as well as using “-omics” tools to examine the molecular mechanisms in depth.

## Figures and Tables

**Table 1:** ANOSIM analyses of the dissimilarities of the biofilm-associated bacterial communities.

Samples		*p*-Value	Samples		*p*-Value	Samples		*p*-Value
A-36 h	B-36 h	1.4 × 10^−^^1^	A-80 h	B-80 h	7.1 × 10^−2^	A-180 h	B-180 h	3.08 × 10^−1^
A-36 h	C-36 h	9.0 × 10^−3^ **	A-80 h	C-80 h	8.0 × 10^−3^ **	A-180 h	C-180 h	1.93 × 10^−1^
A-36 h	D-36 h	8.1 × 10^−2^ *	A-80 h	D-80 h	5.3 × 10^−2^	A-180 h	D-180 h	4.39 × 10^−1^
A-36 h	E-36 h	5.1 × 10^−2^	A-80 h	E-80 h	1.99 × 10^−1^	A-180 h	E-180 h	1.42 × 10^−1^
B-36 h	C-36 h	1.2 × 10^−2^ *	B-80 h	C-80 h	8.2 × 10^−3^ **	B-180 h	C-180 h	6.1 × 10^−2^
B-36 h	D-36 h	9.3 × 10^−2^	B-80 h	D-80 h	5.4 × 10^−2^	B-180 h	D-180 h	7.9 × 10^−2^
B-36 h	E-36 h	9.2 × 10^−2^	B-80 h	E-80 h	5.1 × 10^−2^	B-180 h	E-180 h	6.61 × 10^−1^
C-36 h	D-36 h	1.4 × 10^−2^ *	C-80 h	D-80 h	1.4 × 10^−2^ *	C-180 h	D-180 h	1.3 × 10^−2^ *
C-36 h	E-36 h	8 × 10^−3^ **	C-80 h	E-80 h	1.7 × 10^−2^ *	C-180 h	E-180 h	8 × 10^−3^ **
D-36 h	E-36 h	1.55 × 10^−1^	D-80 h	E-80 h	2.61 × 10^−1^	D-180 h	E-180 h	5.3 × 10^−2^

Note: (A) extracts from *V. alginolyticus* H12, (B) bacterial cultures of *V. alginolyticus* H12, (C) blank control, (D) QSI compound rhodamine isothiocyanate analogue, and (E) positive control furanone, across three time-points (36, 80, and 180 h). * *p* < 0.05 and ** *p* <0.01 indicate significant differences between different groups.

**Table 2:** Physicochemical parameters of seawater.

Time	Temperature (°C)	Salinity (‰)	pH	PO_4_^3−^ (μmol/L)	NO_3_^−^ (μmol/L)	NH_4_^+^ (μmol/L)	TOC (mg/dm^3^)
0 h	26.63	32.45	7.99	1.28	16.77	30.92	2.86
36 h	25.93	32.98	8.01	1.38	15.15	31.43	3.66
80 h	26.59	32.34	8.01	1.72	13.27	28.57	4.97
180 h	24.35	32.33	7.99	1.56	18.63	22.19	4.13

**Table 3:** The major topological features of the association networks. Species correlation network based on the relative abundance of species samples in the OTU (operational taxonomic unit) table. The PC (positive correlation) and NC (negative correlation) represent positive correlation and negative correlation, respectively.

Samples	Average Connectivity	Average Clustering Coefficients	Modularity	Edge	NC	PC
A-36 h	4.56	0.322	0.266	102	44	98
B-36 h	5.03	0.369	0.259	108	21	87
C-36 h	6.52	0.347	0.287	118	8	89
D-36 h	4.76	0.311	0.236	88	10	78
E-36 h	4.77	0.309	0.224	76	21	57
A-80 h	3.71	0.349	0.237	130	52	78
B-80 h	3.98	0.372	0.221	108	30	78
C-80 h	5.58	0.388	0.255	132	42	90
D-80 h	4.29	0.361	0.208	106	48	58
E-80 h	4.12	0.382	0.199	154	70	84
A-180 h	3.11	0.304	0.175	72	38	34
B-180 h	3.25	0.316	0.189	68	24	44
C-180 h	5.69	0.359	0.198	84	34	50
D-180 h	4.09	0.342	0.163	60	20	40
E-180 h	4.52	0.337	0.157	66	28	38

## Supplementary Materials

The following are available online at https://www.mdpi.com/1660-3397/18/9/484/s1. Table S1. Alpha diversity of biofilm microbial community in various groups. The parameter values are presented as the mean ± standard deviation from five measurements. Figure S1. The phenotypic response of biofilm formation subjected to QSI substances. Representative images of biofilm following an 80-h incubation are shown. Signs of biofilm status are divided into three levels, namely, ‘+’, mild biofouling; ‘++’, moderate biofouling; and ‘+++’, severe biofouling. Figure S2. The microbiome structure of bacteria in five treatment groups across three time-points at phylum (A) and family (B) levels. Figure S3. The effect of environmental parameters on microbial community.
