# Supplementary material for: The Rhodamine Isothiocyanate Analogue as a Quorum Sensing Inhibitor Has the Potential to Control Microbially-Induced Biofouling
Source: Mar Drugs. 2020 Sep 22;18(9):484. doi: 10.3390/md18090484 (PMC7551263; doi:10.3390/md18090484)

The rhodamine isothiocyanate analogue as a quorum sensing inhibitor has the potential to control microbe-induced biofouling

Appendix: Supplementary information

Table S1. Alpha diversity of biofilm microbial in various groups. The parameter values are presented as the mean ± standard deviation from five measurements.

Samples	Richness	Chao 1	Shannon	Simpson
A-36h	2847.4±719.6	2849.9±719.42	3.854±0.996	0.3424±0.1536
B-36h	2150.6±279.4	2153.8±278.96	3.332±0.278	0.2848±0.0492
C-36h	2968±577	2970.2±576.38	4.408±0.722	0.189±0.059
D-36h	2739.6±258.4	2742.3±259	3.522±0.668	0.3494±0.2156
E-36h	2674.4±343.6	2676.6±342.92	3.51±0.45	0.3704±0.1416
A-80h	3378.8±929.2	3381.0±929.94	6.302±0.238	0.04628±0.01082
B-80h	3122.2±632.8	3124.7±633.98	5.9±0.34	0.0649±0.0278
C-80h	3620.2±734.8	3622.5±735.1	6.596±0.734	0.0379±0.0126
D-80h	3618.6±976.4	3620.9±977.36	6.028±0.592	0.05584±0.01626
E-80h	3523.2±839.8	3525.4±840.42	6.356±0.414	0.04546±0.02084
A-180h	3960.2±300.8	3962.9±300.3	7.056±0.654	0.03476±0.02204
B-180h	3702.6±247.4	3705.4±247.16	6.794±0.406	0.0468±0.0313
C-180h	3955±815	3957.8±814.32	6.984±0.266	0.03292±0.01368
D-180h	4063.6±275.4	4066.2±274.96	7.336±0.164	0.02354±0.00616
E-180h	3935.8±363.2	3938.6±363.16	7.17±0.23	0.0265±0.0125

Figure S1. The phenotypic response of biofilm formation subjected to QSI substances. The representative images of biofilm following a 80-h incubation were shown. The signs for biofilm status are divided into three levels, namely '+', mild biofouling; '++', moderate biofouling; and '+++', severe biofouling.

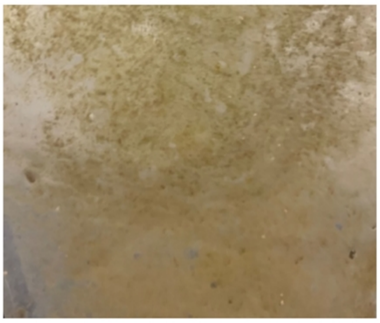

Group A ++
(extracts of strain H12)

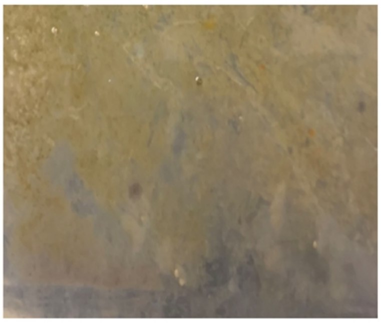

Group B ++
(bacterial cultures of strain H12)

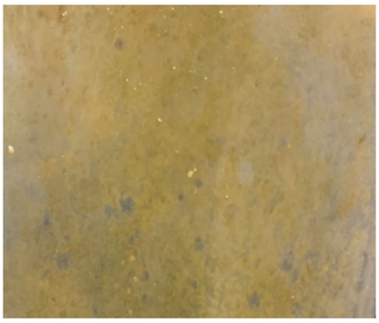

Group C +++
(blank control, seawater only)

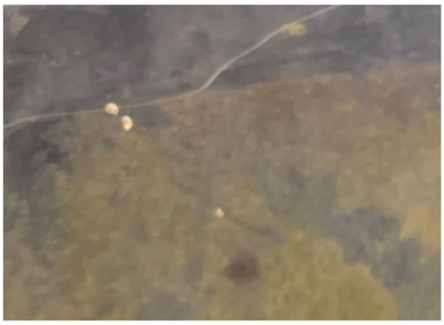

Group D +
(rhodamine isothiocyanate analogue)

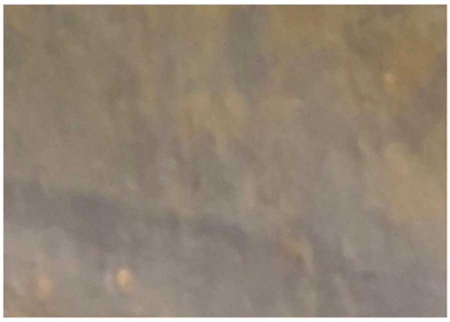

Group E +
Group E (positive control, furanone)

Figure S2. The microbiome structure of bacterial in five treatment groups across three time points at phylum (A) and family (B) levels .

A

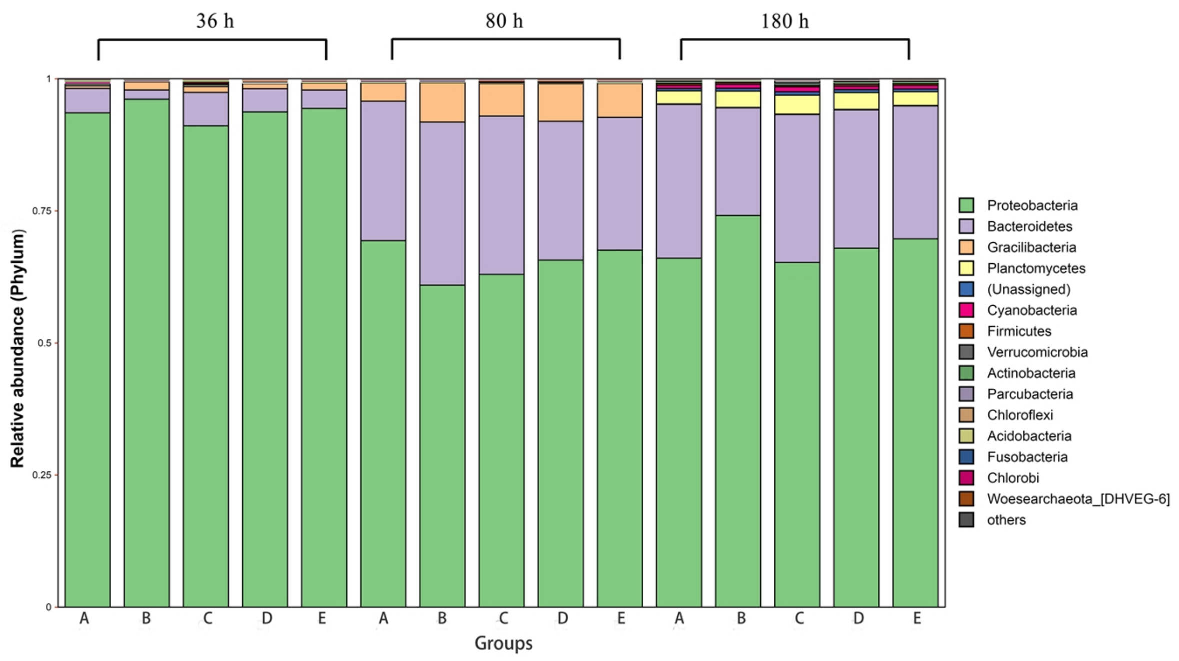

B

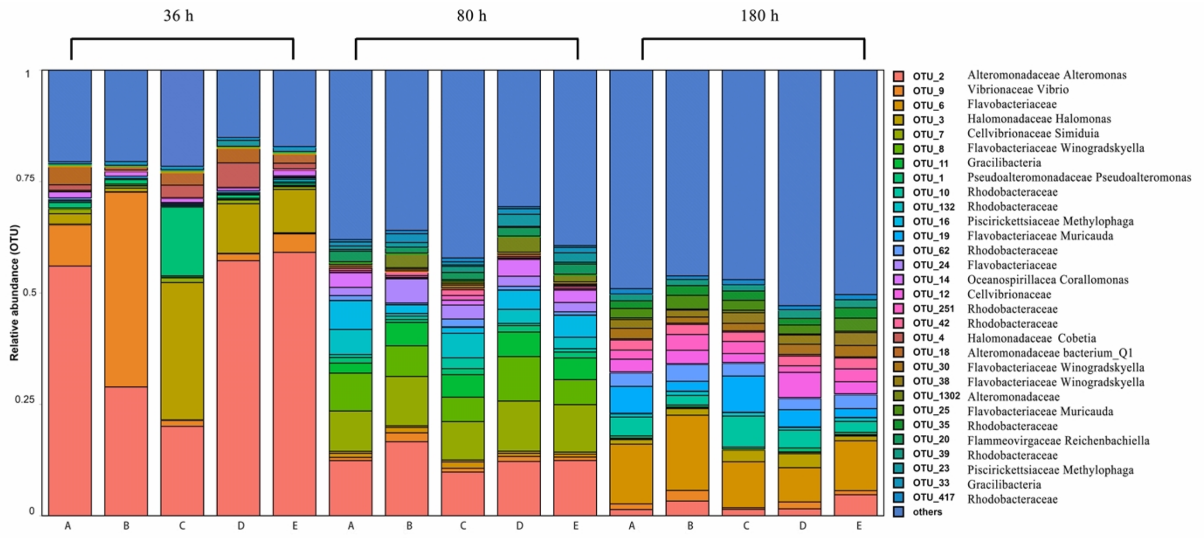

Figure S3. The effect of environmental parameters on microbial community.

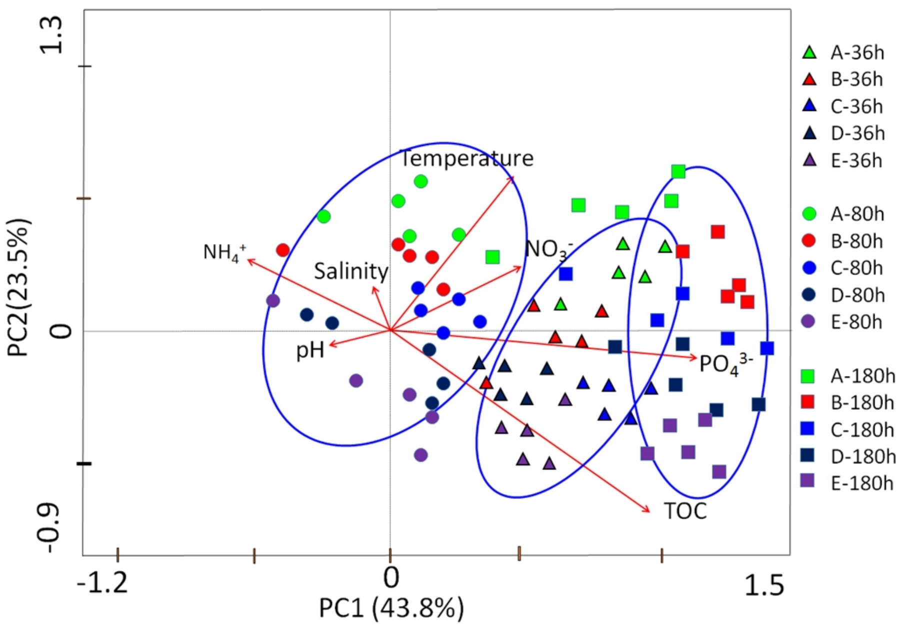

Supplement: Supplementary file 1 [file marinedrugs-18-00484-s001.pdf]
